# Trajectories of sickness absence and disability pension in young working-age adults in Sweden

**DOI:** 10.1038/s41598-025-03739-5

**Published:** 2025-05-28

**Authors:** Iman Alaie, Pia Svedberg, Annina Ropponen, Jurgita Narusyte

**Affiliations:** 1 Division of Insurance Medicine, Department of Clinical Neuroscience, Karolinska Institutet, SE-171 77 Stockholm, Sweden; 2 Department of Psychology, Uppsala University, Uppsala, Sweden; 3 Department of Medical Sciences, Child & Adolescent Psychiatry Unit, Uppsala University, Uppsala, Sweden; 4 Finnish Institute of Occupational Health, Helsinki, Finland

**Keywords:** Epidemiology, Psychiatric disorders, Occupational health, Public health

## Abstract

Sickness absence and disability pension (SADP) constitute a major public health concern, yet the heterogeneity in the developmental trajectories of SADP remains poorly understood. We aimed to identify trajectories of SADP in an observational cohort study of 12,721 Swedish twin individuals, born 1975–1986, who were initially invited to health screening surveys in 2005. Through linkage to nationwide registries, individuals were prospectively followed regarding annual days of SADP from 2006 to 2020. Using group-based trajectory modeling, we identified three distinct SADP trajectories in the total sample: 'high-increasing’ (4%), 'low-increasing’ (13%), and 'low-constant’ (83%). Next, using multinomial logistic regression, we found that mental health-related factors such as neurodevelopmental conditions and common mental disorders were strongly associated with the high-increasing and low-increasing SADP trajectories. Furthermore, increasing age, female sex, low/medium educational level, and blue-collar work were found to be associated with higher odds of belonging to the high-increasing and low-increasing SADP trajectories. We did not find any effect of genetic similarity. Overall, close to every fifth individual showed rising SADP trajectories over time. Research is warranted to address the specific support needs of vulnerable young people struggling with mental health conditions, and to identify any actionable barriers to facilitate labor market integration.

## Introduction

Among young adults under 30 years of age, work incapacity in the form of sickness absence (SA) and disability pension (DP) remains a major public health concern today^1^. This is worrisome for several reasons when viewed from a long-term perspective: young adults have most of their working life ahead of them and the loss of productivity following SA and DP imposes a substantial economic burden on individuals, employers, and societies^1,2^. While a growing body of evidence demonstrates that reduced work capacity is far more common among young adults who are affected by mental disorders than among those who are unaffected^1^, there is still a need to better understand the progression of SA and DP in the early phases of working life. Notably, it is vital from a public health perspective to learn more about the individual factors characterizing those young people at particular risk of SA and DP.

In Sweden, the levels of work incapacity are among the highest reported in Europe^3,4^. Across almost all age groups, mental disorders are the most common diagnostic category among people on SA; in those aged 19–29 years, mental disorders are completely predominant among people receiving DP benefits^5^. Most SA is attributable to common mental disorders (CMDs) such as depressive and anxiety disorders^5^, while most of the DP benefit recipients present with childhood-onset neurodevelopmental conditions such as attention-deficit hyperactivity disorder (ADHD) and autism spectrum disorder (ASD)^6^. Recent nationwide data indicate that mental disorders lead to longer spells of SA than other conditions, and the last decade has witnessed an increase in the duration of SA due to CMDs specifically^5^. Importantly, there is a growing concern that granting SA and DP benefits to young people struggling with mental disorders may trap them in subsequent poverty and welfare dependency in the long run^1,2^. This speaks to the need to advance empirical research into the key factors underlying SA and DP in the young adult population.

Regarding the risk for work incapacity, current literature suggests an important role of a variety of factors, such as sex^7,8^, educational level^9–11^, occupational class^11–13^ and, in particular, mental disorders^1,14^. Also, it is noteworthy that data on twins suggest a moderate genetic contribution to work incapacity^15,16^, with a stronger effect of genetics on SA and DP in young adults below 30 years of age^17^. The important contribution of genetics is also substantiated by numerous studies reporting on estimates of heritability for a variety of psychological traits and mental disorders^18^. These findings underscore the relevance of using genetically informative research designs when addressing associations between individual characteristics (e.g., mental disorders) and work incapacity.

The occurrence of SA by employment sector has gained increasing research interest^13,19–24^, although comparatively less attention has been paid to young adults specifically, with some noteworthy exceptions^25–27^. Previous research has shown a clear increase in all-cause SA rates and sick leave episodes among young female employees in the health and care sectors in Norway and Denmark^25^. Furthermore, recent large-scale research on young employees in Sweden suggests that both employment sector and occupational class may play a role in cause-specific SA granted for CMDs: public sector employees were found to have a higher risk of SA than private sector employees, and blue-collar workers had a higher risk of SA than white-collar workers regardless of employment sector^26^. In a study of young Swedish twins^27^, which was partially based on the same data as the present study, about half of the sample had at least one incident SA spell during a decade-long follow-up from 2006 to 2016, with a somewhat higher proportion among public (53%) than private (42%) sector employees. The same study showed that both private and public sector employees with a history of depression/anxiety symptoms had higher risk of SA than those without such a history; however, the observed association in private sector employees was found to be partially confounded by familial factors^27^. As regards young adults with neurodevelopmental conditions, less research has been undertaken to look at potential differences by employment sector; however, a study on young employees with ADHD suggests no substantial differences between occupational branches concerning the risk of SA and DP^28^.

There is still limited knowledge about the longitudinal patterns of work incapacity among young adults in the general population. Specifically, less is known about the presumed heterogeneity in the development of SA and DP, especially when accounting for both psychiatric diagnoses and symptoms. Here, elevated levels of depression/anxiety symptoms may arguably be of particular relevance to consider, as many people with such ill-health frequently go undiagnosed and untreated^29^. Exploring the longitudinal patterns of work incapacity entails the use of statistical methods applied in latent trajectory analysis, such as group-based trajectory modeling (GBTM)^30,31^, to help identify subpopulations of people who follow similar and distinct developmental trajectories during the time of observation and explore the patterns of variation over time. As a next step, it is possible to relate various individual-level factors (e.g., the presence of ADHD) to each resulting trajectory using standard regression models. This approach may provide a deeper understanding of the longitudinal patterns of SA and DP, which may be helpful to better identify and support vulnerable groups at particular risk of future work incapacity. Gaining deeper insights into some of the potential key factors involved in how work incapacity develops and unfolds over time could be an important step toward promoting increased health equity and labor market participation^32^. This may be key to help improve evidence-based policies and interventions that broadly target any actionable factors underlying the deterioration of health and functioning of youths and young adults.

This genetically informative study aimed to identify trajectories of work incapacity in young working-age adult twins of the general population, accounting for a host of individual characteristics such as mental health conditions, sociodemographic factors, and occupational class. We also sought to identify trajectories of work incapacity by employment sector.

## Methods

### Study design and population

This was an observational cohort study based on extensive longitudinal data gathered as part of the Swedish Twin project Of Disability pension and Sickness absence (STODS)^33^. All twins were born in Sweden and were originally eligible for participation in either of two health screening survey studies conducted by the Swedish Twin Registry; the Twin study of CHild and Adolescent Development (TCHAD)^34^ and the Study of Twin Adults: Genes and Environment (STAGE)^35^, respectively. Both TCHAD and STAGE were completed around the year 2005, which was set as the baseline in the present study.

This study included a combination of measures from survey- and registry-based data. Annual deidentified data were obtained from nationwide population-based registries kept by various government agencies and were linked at the individual level to the participants using the unique personal identity number assigned to all Swedish residents^36^. These registry-based data were available both retrospectively and prospectively in relation to the baseline of the study in 2005, with a total follow-up period extending over 15 years from 2006 to 2020.

The source population of TCHAD included twins who were born in the years 1985–1986 (n = 2960). Twins and/or their parents were contacted on four waves of measurement, when the twins were aged 8–9, 13–14, 16–17, and 19–20 years. At each wave, the twins/parents were surveyed using a comprehensive battery of questionnaires on physical health as well as emotional and behavioral problems. Details about the study design, measures, and response rates have been published elsewhere^34^. In the present study, 2844 twin individuals from TCHAD were included.

The source population of STAGE included twins who were born between the years 1959 and 1985 (n = 42,582), when those eligible were aged 20–46 years. The twins were invited to take part in a survey with self-report questionnaires on for example various aspects of health and lifestyle. Details about the study design, measures, and response rate have been published elsewhere^35^. In the present study, 9877 twin individuals from STAGE were included as participants.

Here, we collapsed the data collected in TCHAD and STAGE, but only for STAGE participants who were aged 30 years or younger at the time of the survey completion. The final analytic sample of participants comprised 12,721 twin individuals, of which 5239 were monozygotic (MZ), 3379 were dizygotic same-sexed (DZSS), 3563 were dizygotic opposite-sexed (DZOS), and 540 had unknown or missing data on zygosity.

The ethical vetting was performed and approved by the Regional Ethical Review Board of Stockholm, Sweden (Dnr: 2007/524–31, 2010/1346–32/5, 2014/311–32, 2015/1809–32, 2017/128–32), and the Swedish Ethical Review Authority (Dnr: 2021–03482). The study has been performed in accordance with the ethical standards laid down in the 1964 Declaration of Helsinki and its later amendments. Informed consent was obtained from all individuals participating in STAGE and TCHAD (or their parents or legal guardians). The participants in STODS have previously been informed about registry-based linkages^27^.

### Exposure variables

Mental health-related factors were the main exposures of interest. Consistent with other research using registry-based data^37^, *ADHD* was coded as a binary variable (yes vs. no), which was defined as a lifetime clinical diagnosis of ADHD (ICD-10: F90) and/or a lifetime dispensation of medication for ADHD, including amphetamines (ATC: N06BA01, N06BA02), methylphenidate (ATC: N06BA04), or atomoxetine (ATC: N06BA09). Here, lifetime ADHD refers to any such diagnosis or medication registered either before or during follow-up. This information was based on data obtained from the National Patient Registry (NPR) and the Prescribed Drug Registry (PDR), respectively, kept by the National Board of Health and Welfare. The NPR includes information on somatic and psychiatric inpatient care from 1973 onwards and specialized outpatient care from 2001 onwards. The NPR includes diagnostic codes recorded at visits to both public and private caregivers, with nearly 100% coverage for inpatient care and 90% coverage for outpatient care. Furthermore, the PDR includes information on all dispensed medication across all pharmacies in Sweden since July 2005, with good coverage overall.

*Other neurodevelopmental condition* was coded as a binary variable (yes vs. no), defined as a lifetime clinical diagnosis of language disorder, specific learning disorder, developmental coordination disorder, autism spectrum disorder, and/or tic disorder (ICD-10: F80-F89, F95). This information was based on data obtained from the NPR.

*Common mental disorder* was coded as a binary variable (yes vs. no), defined as a clinical diagnosis of depressive, anxiety, and/or stress-related disorder in 2001–2005 (ICD-10: F32-F33, F34.1, F34.9, F40-F43). This information was based on data obtained from the NPR.

*Depression and/or anxiety symptoms* were coded as a binary variable (yes vs. no) and were measured through self-report questionnaires administered at age 19–20 years in TCHAD or ages 18–30 years in STAGE. In TCHAD, depression/anxiety symptoms were assessed with the Child Behavior Checklist (CBCL) using a three-point Likert scale^38^. Permission to use the CBCL was obtained prior to the initiation of TCHAD. In this study, exposure classification was based on the Internalizing scale, subsuming the Anxious-Depressed, Withdrawn-Depressed, and Somatic Complaints subscales. An adapted version of the questionnaire (i.e., Young Adult Self-Report) was used to classify cases, defined as ≥ 65 T-scores on the Internalizing scale. In STAGE, lifetime history of major depressive disorder (MDD) and generalized anxiety disorder (GAD) was measured using the computerized Composite International Diagnostic Interview–Short Form (CIDI-SF)^39^. The CIDI-SF was modified from its original design for 12-month prevalence to assess the lifetime prevalence of MDD and GAD, respectively.

### Covariate variables

*Age* at baseline was coded as a continuous variable (years).

*Sex* was coded as a binary variable (female vs. male).

*Educational level* was coded as a binary variable (low-to-medium with < 14 years vs. high with ≥ 14 years), reflecting the highest level of education achieved across the follow-up (i.e., 2006–2020). This information was based on data from the Longitudinal Integration Database for Health Insurance and Labor Market Studies (LISA) kept by Statistics Sweden^40^. LISA started in 1990 and is updated each year by the transmission of annual data on labor market participation, educational systems, and social sectors from various source registries. The LISA database includes all individuals (aged ≥ 16 or, more recently, 15 years) who were registered as residents in Sweden as of December 31 each year.

*Occupational class* was coded as a binary variable (blue- vs. white-collar), which was determined using the Standard for Swedish Occupational Classification (SSYK) for identifying various types of occupations based on data from LISA. The SSYK system has several occupational categories, which were dichotomized into blue-collar and white-collar workers in line with a simplified version of the classification by Thell^41^ based on what the work entails, the educational qualification required, and supervision responsibility. The blue-collar category encompasses service workers and shop sale workers; skilled agricultural and fishery workers; craft and related trades workers; plant and machine operators and assemblers; and elementary occupations. The white-collar category encompasses legislators, senior officials, and managers; professionals, technicians, and associate professionals; and clerks. Participants were classified as being blue- or white-collar workers after maintaining employment with either type of collar color for at least three years in total during the follow-up (i.e., 2006–2020). If a participant had held positions with both collar colors for an equal number of years, the most recent one by the end of follow-up was assigned.

*Zygosity* was coded as a categorical variable with four levels (MZ; DZSS; DZOS; unknown), based on information obtained from the Swedish Twin Registry.

### Stratification variable

*Employment sector* was determined through stratification of the sample by the main sector of gainful employment, in terms of private vs. public sector, using data from LISA. Private sector employees were those employed in private companies or organizations, while public sector employees were those employed by national authorities, county councils, or municipalities. We classified participants as being private or public sector employees depending on whether they had been employed in either sector for a minimum of three years during follow-up (i.e., 2006–2020). If a participant had worked in both sectors for an equal number of years, the most recent sector by the end of follow-up was assigned^42^.

### Outcome variable

The outcome of interest was SA and DP (hereafter referred to as *SADP*). This was an aggregate outcome variable, including consecutive annual net days of SA and/or DP spanning from 2006 to 2020 (range 0–365 days). In Sweden, part-time (25%, 50%, or 75%) or full-time (100%) SA or DP can be granted. There is also the possibility to be granted both part-time SA and part-time DP simultaneously. The net days of SA and DP were therefore calculated: e.g., 2 gross days of 50% SA or DP were equal to 1 net day of SADP.

In Sweden, SA can be granted to all residents who have an income from work or unemployment benefits if there is a reduced work capacity due to disease or injury. For the initial 14 days of a sick-leave spell, an employee receives sick pay from the employer, though there is one qualifying day (more for self-employed) without any pay. If the work capacity is still reduced after 14 days, the employee can apply for SA benefits from the Swedish Social Insurance Agency (SSIA). Sick payments correspond to approximately 80% of lost income and presuppose work prior to the benefit claims.

Furthermore, residents who have a permanently impaired work capacity are entitled to DP benefits. Since 2003, however, only temporary activity benefits can be granted to young individuals aged 19–30 years, whereas individuals aged 30–64 can be entitled to permanent disability benefits. DP amounts to a capped 65% of lost income, with reduced benefits for those who have not worked previously or have had a low income. Any claims for SA and DP benefits are first preceded by medical assessments made by certified physicians and, thereafter, handled by SSIA.

The outcome data were obtained from the Micro Data for the Analysis of Social Insurance (MiDAS) database, held by SSIA, covering the entire Swedish population.

### Statistical analysis

Statistical analyses were performed in three sequential steps, both for the total analytic sample (n = 12,721) and, if participants had entered the workforce, stratified by employment sector in terms of private (n = 7437) and public (n = 4378) sector.

First, we calculated descriptive statistics of the analytic sample, summarizing the number and proportion of participants with regard to individual characteristics.

Second, we used GBTM^30^ to identify groups or clusters of participants who follow distinct developmental trajectories of SADP over the course of follow-up. We fitted a zero-inflated Poisson model to the annual SADP data, given that an excess of zero counts in SADP days was observed at each follow-up year. Here, only SADP days were entered in the estimation process. The optimal number of trajectory groups (or clusters) and the degrees of the polynomial function were determined using domain knowledge as well as model fit statistics, including the Bayesian Information Criterion (BIC) and the Akaike Information Criterion (AIC), along with the average posterior probability of group membership (APPA) and the odds of correct classification (OCC). We considered the size of the estimated trajectory groups when selecting the final solution to ensure adequate statistical power in the subsequent multinomial logistic regression analysis. Therefore, a three-group solution with at least 4% of the study population in each trajectory group was preferred to a four-group solution. After selecting the best-fitting and most parsimonious model, the posterior probabilities of belonging to each latent trajectory group were computed and, consequently, participants were assigned to the trajectory group for which they had the highest posterior probability. An APPA of ≥ 70 was used as an indication of goodness of fit, and an OCC of ≥ 5.0 was deemed indicative of high assignment accuracy^30^.

Third, the associations between covariates and SADP trajectories were examined by multinomial logistic regression models using the 'low-constant’ trajectory group (with the least SADP) as the reference throughout all analyses. All covariates were entered simultaneously into the regression models and any between-groups difference was estimated as an Odds Ratio (OR) with a 95% Confidence Interval (CI) including adjusted standard errors to account for the clustered nature of the data (i.e., twin pairs). Statistical significance was set at *p* < 0.05. A log-likelihood test was used to test differences between trajectory groups regarding all covariates. McFadden’s pseudo-R^2^ was used to evaluate the strength of these associations, i.e., how much the included covariates were able to explain the total variance. Differences in pseudo-R^2^ were calculated for each covariate by consecutively excluding one covariate from the full model.

We observed two sources of missingness in the registry-based follow-up data: death (n = 86) and resettling abroad (n = 751). The mean total time of missingness in the registries among those who had emigrated was 6.5 years (SD 4.8). Data management and analyses were performed using SAS version 9.4, Stata version 16.1, and R version 4.2.3. GBTM analyses were performed using the crimCV package^43^.

## Results

Descriptive characteristics are presented in Table 1. The mean age of participants at the start of follow-up was 24.0 years (SD 3.7) in the total sample. Fifty-five percent of participants were female in the total sample, with female employees being in majority in the public (73%) and male employees being in majority in the private (56%) sector. In the total sample, 54% had achieved a high educational level, and 46% a low/medium educational level. While most public sector employees had a high educational level (70% vs. 30%), the proportions among private sector employees were roughly balanced (47% vs. 53%).

**Table 1:** Characteristics of young working-age adults, shown for the total sample and by main sector of employment.

Characteristics	Total sample (n = 12,721)		Private sector employees (n = 7437)		Public sector employees (n = 4378)	
	n	%	n	%	n	%
Age, mean (SD)^a^	24.0	3.7	23.9	3.7	24.2	3.7
Sex						
Female	7002	55.0	3267	43.9	3210	73.3
Male	5719	45.0	4170	56.1	1168	26.7
Educational level^b^						
High (≥ 14 y)	6844	53.8	3506	47.1	3072	70.2
Low/medium (< 14 y)	5877	46.2	3931	52.9	1306	29.8
Collar color^c^						
White collar	6501	51.1	3552	47.8	2808	64.1
Blue collar	5699	44.8	3820	51.4	1560	35.6
Missing information	521	4.1	65	0.9	10	0.2
ADHD diagnosis^d^						
No	12,392	97.4	7278	97.9	4286	97.9
Yes	329	2.6	159	2.1	92	2.1
Other neurodevelopmental diagnosis^e^						
No	12,569	98.8	7384	99.3	4350	99.4
Yes	152	1.2	53	0.7	28	0.6
Common mental disorder diagnosis^f^						
No	12,372	97.3	7300	98.2	4245	97.0
Yes	349	2.7	137	1.8	133	3.0
Depression/anxiety symptoms^g^						
No	7938	62.4	4733	63.6	2797	63.9
Yes	1444	11.4	678	9.1	596	13.6
Missing information	3339	26.2	2026	27.2	985	22.5
Sickness absence/disability pension^h^						
No days	11,680	91.8	6943	93.4	4032	92.1
1–180 days	713	5.6	402	5.4	278	6.3
> 180 days	328	2.6	92	1.2	68	1.6
Zygosity						
Monozygotic	5239	41.2	3048	41.0	1836	41.9
Dizygotic same-sexed	3379	26.6	1949	26.2	1200	27.4
Dizygotic opposite-sexed	3563	28.0	2122	28.5	1161	26.5
Unknown/missing	540	4.2	318	4.3	181	4.1

^a^Age at first year of follow-up; ^b^Highest level of education; ^c^Main designation; ^d^Attention-deficit/hyperactivity disorder; ^e^Autism spectrum disorder, language disorder, or tic disorder; ^f^Depressive, anxiety, or stress-related disorder; ^g^Self-ratings from health screening surveys; ^h^Sickness absence and/or disability pension at first year of follow-up.

### Identification of latent trajectories of sickness absence and disability pension

In the total sample, the model fit statistics indicated the superiority of a four-group solution as implied by the BIC and AIC values as well as the APPA and OCC (Supplementary Tables S1-S3). After considering the most parsimonious model and assessing the relative changes in model fit depending on the polynomial orders used, we decided on a three-group model with cubic polynomials based on our criteria for model selection. Likewise, after stratification of the total sample by employment sector, we decided on a three-group model applied to both the private and the public sector employees.

**Fig. 1:**
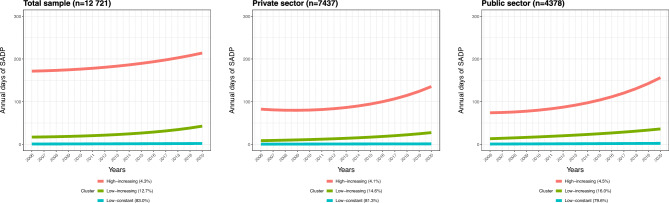
Trajectories of sickness absence and disability pension (SADP) among young Swedish adults, shown for the total sample and stratified by employment sector.

The results of the final GBTM analysis are shown in Fig. 1, illustrating the counts of the annual net days of SADP while accounting for the zero-inflation process. In the total sample, the trajectories of SADP were labeled according to their visual shapes: ‘High-increasing’ (4.3%), ‘Low-increasing’ (12.7%), and ‘Low-constant’ (83.0%). For private sector employees, the trajectories of SADP were labeled: ‘High-increasing’ (4.1%), ‘Low-increasing’ (14.6%), and ‘Low-constant’ (81.3%). For public sector employees, the trajectories of SADP were labeled ‘High-increasing’ (4.5%), ‘Low-increasing’ (16.0%), and ‘Low-constant’ (79.6%).

### Associations of individual factors with trajectories of sickness absence and disability pension

For the total sample, the results of the multinomial logistic regression analysis are provided in Table 2. Virtually all estimates from this analysis were statistically significant. For example, those affected by CMDs had higher odds of belonging to the high-increasing (OR = 4.87, 95% CI = 3.33–7.13) and low-increasing (OR = 2.32, 95% CI = 1.75–3.08) trajectories of SADP, compared with those unaffected.

**Table 2:** Results from multinomial logistic regression based on the total sample with SADP trajectories regressed on covariates using the low-constant trajectory (n = 10,588) as reference.

Covariates	High-increasing (n = 545)		Low-increasing (n = 1588)			
	OR (95% CI)	*p*	OR (95% CI)	*p*	Log-likelihood test (*p*-value)	R^2^ difference
Age	1.06 (1.03–1.09)	< 0.001	1.04 (1.02–1.05)	< 0.001	36.51 (< 0.001)	0.003
Sex						
Women (Ref: Men)	1.79 (1.44–2.23)	< 0.001	2.03 (1.80–2.28)	< 0.001	164.48 (< 0.001)	0.012
Educational level						
Low/medium (Ref: High)	3.73 (2.77–5.02)	< 0.001	1.37 (1.20–1.58)	< 0.001	110.73 (< 0.001)	0.008
Collar color						
Blue (Ref: White)	1.99 (1.47–2.68)	< 0.001	1.52 (1.33–1.75)	< 0.001	342.47 (< 0.001)	0.025
Missing (Ref: White)	11.02 (7.85–15.46)	< 0.001	0.24 (0.13–0.44)	< 0.001		
ADHD diagnosis						
Yes (Ref: No)	6.21 (4.10–9.40)	< 0.001	4.25 (3.14–5.75)	< 0.001	144.12 (< 0.001)	0.010
Other neurodevelopmental diagnosis						
Yes (Ref: No)	17.27 (10.06–29.66)	< 0.001	2.31 (1.27–4.21)	0.006	150.06 (< 0.001)	0.011
Common mental disorder diagnosis						
Yes (Ref: No)	4.87 (3.33–7.13)	< 0.001	2.32 (1.75–3.08)	< 0.001	87.07 (< 0.001)	0.006
Depression/anxiety symptoms						
Yes (Ref: No)	3.56 (2.71–4.68)	< 0.001	1.96 (1.67–2.29)	< 0.001	138.09 (< 0.001)	0.010
Missing (Ref: No)	1.77 (1.39–2.25)	< 0.001	1.27 (1.12–1.45)	< 0.001		
Zygosity						
Monozygotic (Ref: Dizygotic same-sex)	1.22 (0.92–1.61)	0.171	1.08 (0.94–1.24)	0.278	15.60 (0.016)	0.001
Dizygotic opposite-sex (Ref: Dizygotic same-sex)	1.64 (1.25–2.16)	< 0.001	1.08 (0.93–1.25)	0.319		
Unknown (Ref: Dizygotic same-sex)	1.38 (0.83–2.31)	0.216	1.18 (0.90–1.56)	0.224		

McFadden’s Pseudo R^2^ for full model = 0.136

For private sector employees, the results of the multinomial logistic regression analysis are provided in Table 3. Most estimates from this analysis were statistically significant. For example, those affected by CMDs had higher odds of belonging to the high-increasing (OR = 3.68, 95% CI = 2.16–6.26) and low-increasing (OR = 2.29, 95% CI = 1.48–3.53) trajectories of SADP, compared with those unaffected.

**Table 3:** Results from multinomial logistic regression based on private sector employees with SADP trajectories regressed on covariates using the low-constant trajectory (n = 6097) as reference.

Covariates	High-increasing (n = 303)		Low-increasing (n = 1037)			
	OR (95% CI)	*p*	OR (95% CI)	*p*	Log-likelihood test (*p*-value)	R^2^ difference
Age	1.06 (1.02–1.09)	0.001	1.03 (1.01–1.05)	0.003	16.88 (< 0.001)	0.002
Sex						
Women (Ref: Men)	2.05 (1.59–2.65)	< 0.001	2.08 (1.81–2.39)	< 0.001	127.65 (< 0.001)	0.015
Educational level						
Low/medium (Ref: High)	2.53 (1.81–3.52)	< 0.001	1.64 (1.39–1.94)	< 0.001	65.05 (< 0.001)	0.008
Collar color						
Blue (Ref: White)	2.06 (1.49–2.85)	< 0.001	1.53 (1.30–1.82)	< 0.001	57.61 (< 0.001)	0.007
Missing (Ref: White)	4.51 (2.10–9.66)	< 0.001	0.37 (0.11–1.24)	0.108		
ADHD diagnosis						
Yes (Ref: No)	7.62 (4.65–12.48)	< 0.001	3.48 (2.27–5.34)	< 0.001	75.71 (< 0.001)	0.009
Other neurodevelopmental diagnosis						
Yes (Ref: No)	5.67 (2.33–13.79)	< 0.001	1.63 (0.71–3.76)	0.250	18.83 (< 0.001)	0.002
Common mental disorder diagnosis						
Yes (Ref: No)	3.68 (2.16–6.26)	< 0.001	2.29 (1.48–3.53)	< 0.001	28.34 (< 0.001)	0.003
Depression/anxiety symptoms						
Yes (Ref: No)	3.82 (2.75–5.31)	< 0.001	1.84 (1.49–2.29)	< 0.001	90.08 (< 0.001)	0.011
Missing (Ref: No)	1.93 (1.44–2.58)	< 0.001	1.42 (1.21–1.66)	< 0.001		
Zygosity						
Monozygotic (Ref: Dizygotic same-sex)	1.32 (0.94–1.85)	0.107	1.09 (0.92–1.29)	0.338	11.17 (0.083)	0.001
Dizygotic opposite-sex (Ref: Dizygotic same-sex)	1.62 (1.15–2.27)	0.006	0.97 (0.81–1.16)	0.737		
Unknown (Ref: Dizygotic same-sex)	1.56 (0.90–2.70)	0.116	0.93 (0.66–1.33)	0.699		

McFadden’s Pseudo R^2^ for full model = 0.085

For public sector employees, the results of the multinomial logistic regression analysis are provided in Table 4. Several estimates from this analysis were statistically significant. For example, those affected by CMDs had higher odds of belonging to the high-increasing (OR = 4.23, 95% CI = 2.31–7.72) and low-increasing (OR = 2.93, 95% CI = 1.93–4.43) trajectories of SADP, compared with those unaffected.

**Table 4:** Results from multinomial logistic regression based on public sector employees with SADP trajectories regressed on covariates using the low-constant trajectory (n = 3490) as reference.

Covariates	High-increasing (n = 196)		Low-increasing (n = 692)			
	OR (95% CI)	*p*	OR (95% CI)	*p*	Log-likelihood test (*p*-value)	R^2^ difference
Age	1.04 (0.99–1.08)	0.069	1.04 (1.01–1.06)	0.002	11.84 (0.003)	0.002
Sex						
Women (Ref: Men)	1.69 (1.16–2.45)	0.006	2.16 (1.72–2.70)	< 0.001	55.55 (< 0.001)	0.010
Educational level						
Low/medium (Ref: High)	1.52 (1.00–2.31)	0.048	1.25 (0.98–1.58)	0.069	6.42 (0.040)	0.001
Collar color						
Blue (Ref: White)	2.45 (1.60–3.75)	< 0.001	1.41 (1.12–1.77)	0.003	23.84 (< 0.001)	0.004
Missing (Ref: White)	NA	NA	1.91 (0.39–9.38)	0.424		
ADHD diagnosis						
Yes (Ref: No)	9.38 (4.93–17.83)	< 0.001	3.45 (2.03–5.87)	< 0.001	55.01 (< 0.001)	0.010
Other neurodevelopmental diagnosis						
Yes (Ref: No)	15.93 (3.91–64.93)	< 0.001	4.53 (1.41–14.49)	0.011	24.98 (< 0.001)	0.005
Common mental disorder diagnosis						
Yes (Ref: No)	4.23 (2.31–7.72)	< 0.001	2.93 (1.93–4.43)	< 0.001	37.21 (< 0.001)	0.007
Depression/anxiety symptoms						
Yes (Ref: No)	2.77 (1.86–4.12)	< 0.001	1.68 (1.33–2.13)	< 0.001	36.85 (< 0.001)	0.007
Missing (Ref: No)	1.45 (0.99–2.12)	0.056	1.09 (0.89–1.35)	0.400		
Zygosity						
Monozygotic (Ref: Dizygotic same-sex)	1.33 (0.88–2.01)	0.180	1.09 (0.88–1.34)	0.456	7.78 (0.255)	0.001
Dizygotic opposite-sex (Ref: Dizygotic same-sex)	1.61 (1.05–2.47)	0.030	1.16 (0.92–1.47)	0.198		
Unknown (Ref: Dizygotic same-sex)	1.99 (0.98–4.00)	0.055	1.31 (0.84–2.03)	0.234		

McFadden’s Pseudo R^2^ for full model = 0.077

## Discussion

This population-based longitudinal twin study sought to identify latent trajectories of SADP among young Swedish adults. In the total sample, we identified three distinct group-based trajectories of SADP across the follow-up period. We also identified three distinct SADP trajectories among private and public sector employees, respectively, where the shapes of trajectories were very much alike throughout follow-up. The vast majority followed the low-constant SADP trajectories, estimated as making up about 83% of the total sample, 81% of the private sector, and 80% of the public sector. About 4% followed the high-increasing SADP trajectories, with the most salient difference observed in the total sample, where the gap between the high-increasing and the other trajectories was most pronounced. Although discernible, the gaps between the corresponding trajectories among both private and public sector employees were of a somewhat lesser magnitude. This may indeed reflect the heterogeneity of the study population in terms of individual-level characteristics, as the total sample comprised the full range of participants from the general population of young adult Swedish twins. This includes, for example, those who entered and remained in the labor force throughout the follow-up as well as those who had no or poor labor market attachment early on, most likely due to their medical conditions and associated functional impairments. Hence, a proportion of those with a severely reduced work capacity were only included in the total sample analysis. This also explains why some 4% of the total sample had missing data on collar color.

Mental health-related factors, in particular, were found to be associated with less favorable outcomes, especially the high-increasing SADP trajectories. For example, those diagnosed with ADHD had higher odds of belonging to the high-increasing SADP trajectories, with ORs > 6, and higher odds of belonging to the low-increasing SADP trajectories, with ORs > 3. The magnitudes of associations were more varying for those diagnosed with other neurodevelopmental conditions, with ORs ranging from 1 to 17, but not consistently statistically significant across all comparisons performed. Furthermore, those diagnosed with CMDs and those with self-reported depression/anxiety symptoms also showed increased odds of belonging to the less favorable SADP trajectories, particularly the high-increasing SADP trajectories. Altogether, our findings add to a growing body of observational evidence suggesting that those affected by mental disorders at a young age are at risk of future work incapacity^27,37,44–47^. Importantly, however, our study contributes to current literature by way of design, insofar as prior work in this area has been designed as either regular epidemiologic studies estimating the risk of SADP by comparing cases (e.g., those with ADHD) to controls (e.g., those without ADHD)^45^, or as studies applying GBTM to clinical cohorts^42,48^. Here, we targeted the general population of young working-age adults when first exploring the trajectories of SADP, and then examining the role of various individual factors associated with the identified SADP trajectories. This may complement our understanding of how SADP continuously develops over time in the general population of young adults while also taking into account the simultaneous role of several important individual factors. More importantly, our findings lend support to national policies and programs designed to help identify and address the individual needs of at-risk youths, such as pupils struggling with, for example, neurodevelopmental disorders. Such timely and targeted interventions aiming to promote a successful school-to-work transition have in recent years shown promising results in, for example, Sweden^49^, further underscoring the importance of well-coordinated efforts by relevant authorities and municipalities to help prevent long-term labor market marginalization in this group.

Participants with missing data on self-rated depression/anxiety, following nonresponse or attrition in the health screening surveys, were found to have higher odds of belonging to the less favorable SADP trajectories in both the total sample and the private sector. A proportion of those may presumably have had a history of various health issues, such as mental ill-health: we noticed that about 3% of those with such missingness had been diagnosed with CMDs in specialized outpatient or inpatient care in 2001–2005 (data not shown). Here, the use of two different but related measures, one based on self-ratings and one based on clinical diagnoses, may be considered as partially complementary to one another, though there are several methodologically important differences (e.g., time-frames assessed, diagnostic validity, coverage of probable cases across the full spectrum of symptom severity). Nonetheless, this approach may help to enhance the exposure classification of cases with more severe CMDs, notably for those suffering from such severe depression/anxiety symptoms impacting on their ability to take part in surveys.

In terms of factors other than mental health, we found that having higher age, female sex, and low/medium education were generally associated with the less favorable SADP trajectories, even after adjustment for multiple individual-level indicators such as mental health conditions. This pattern of results is in line with research findings from previous studies^8,9^. Furthermore, we observed that blue-collar workers had higher odds of belonging to the less favorable trajectories of SADP as compared with white-collar workers. This is also consistent with other research suggesting adverse outcomes in working life among blue-collar workers^11–13,26^. Again, the specific estimates should be interpreted in consideration of the other covariates included in the statistical model. Moreover, we adjusted for zygosity to account for the role of genetic similarity for the resulting trajectory group memberships and found that dizygotic opposite-sexed twins had higher odds of belonging to the high-increasing SADP trajectories across all analyses. While this may potentially mirror the influence of sex rather than any potential genetic effects, we were not able to disentangle sex-specific and genetic contributions to the trajectory memberships using the present GBTM framework.

### Strengths and limitations

This study had a relatively large sample and a distinct combination of data from screening surveys and high-quality nationwide registries. A particularly noteworthy strength is the use of the nationwide Swedish registries, which are free from various reporting biases and have virtually no loss to follow-up. The long follow-up period spanning 15 years enhanced the chances to capture any temporal changes in the development of work incapacity over time. Overall, our results should apply to the general population of young working-age adults as Swedish twins are representative of the general population^50^; but, our results may not be representative of other populations or age-cohorts. However, despite the large sample size and determined zygosity, we did not find any effect of genetic similarity. Research using more sophisticated methods to evaluate genetic influences would be merited to shed further light on this. Nonetheless, the use of twins from this large sample can be considered a strength, as these results add to the literature on unrelated individuals. A limitation of the study is the proportion of participants with missing survey data on depression/anxiety symptoms, especially if those with such symptoms were more likely to be among the nonresponders of the surveys than those without symptoms. Another limitation is that we could not address the role of psychosocial factors at the workplace that may be linked to the incidence and levels of SADP over time^51^. Finally, we acknowledge that the study was somewhat statistically underpowered to reliably detect potential differences in outcomes regarding some individual-level indicators, especially those with a low prevalence. The rather imprecise estimates should thus be interpreted cautiously.

## Conclusion

This study provided further knowledge about the heterogeneity in the longitudinal patterns of work incapacity among young working-age adults. Close to every fifth participant showed rising trajectories of work incapacity over time. Mental health-related factors emerged as the strongest predictors of the less favorable SADP trajectories. This highlights the importance of increasing our understanding on how best to support young individuals struggling with mental health conditions as they make the transition from adolescence to adulthood. Research is warranted to address the specific support needs of young individuals vulnerable to marginalization from the labor market, and to identify any actionable barriers to facilitate labor market integration.

## Supplementary Information

Supplementary Information.

## Data Availability

The data that support the findings of this study are available from the original sources: the Swedish Twin Registry, Statistics Sweden, Swedish Social Insurance Agency, and the National Board of Health and Welfare. Restrictions apply to the availability of the data used in this study based on the Swedish Twin project Of Disability pension and Sickness absence (STODS), which were used with ethical permission for the current study and therefore are not publicly available. According to the General Data Protection Regulation, the Swedish law SFS 2018:218, the Swedish Data Protection Act, the Swedish Ethical Review Act, and the Public Access to Information and Secrecy Act, this type of sensitive data can be made available only after legal review, for researchers who meet the criteria for access to this type of sensitive and confidential data. Readers may contact the last author regarding the details.
