# Supplementary material for: Trajectories of sickness absence and disability pension in young working-age adults in Sweden
Source: Sci Rep. 2025 May 28;15:18592. doi: 10.1038/s41598-025-03739-5 (PMC12117053; doi:10.1038/s41598-025-03739-5)
Supplement: Supplementary file 1 — Supplementary Information. [file 41598_2025_3739_MOESM1_ESM.docx]

**Supplementary Information**

Trajectories of sickness absence and disability pension in young working-age adults in Sweden

| **Table of content** | **Page #** |
| --- | --- |
| Table S1. Model adequacy tests for the total sample | 2 |
| Table S2. Model adequacy tests for private sector employees | 2 |
| Table S3. Model adequacy tests for public sector employees | 2 |

| Table S1. Model adequacy tests for the total sample (n=12 721) | | | | | | |
| --- | --- | --- | --- | --- | --- | --- |
| **Model selection**  **BIC / AIC** | **Group** | **n (%)** | **Posterior probability of group membership** | **Odds of correct classification** | **Probability observed** | **Probability expected** |
| **2-group** | 1 | 913 (7.2) | 99.8 | 6726.6 | 7.2 | 7.2 |
| BIC=1426884.8 | 2 | 11808 (92.8) | 100.0 | 491.2 | 92.8 | 92.8 |
| AIC=1426788.0 |  |  |  |  |  |  |
|  |  |  |  |  |  |  |
| **3-group** | 1 | 545 (4.3) | 99.7 | 7919.4 | 4.3 | 4.3 |
| BIC=1151161.6 | 2 | 1588 (12.7) | 99.5 | 1269.4 | 12.5 | 12.7 |
| AIC=1151020.1 | 3 | 10588 (83.0) | 99.6 | 57.8 | 83.2 | 83.0 |
|  |  |  |  |  |  |  |
| **4-group** | 1 | 388 (3.0) | 99.6 | 8847.5 | 3.1 | 3.0 |
| BIC=1050510.3 | 2 | 654 (5.2) | 99.4 | 2862.1 | 5.1 | 5.2 |
| AIC=1050324.0 | 3 | 1796 (14.6) | 98.9 | 540.9 | 14.1 | 14.6 |
|  | 4 | 9883 (77.1) | 99.1 | 34.3 | 77.7 | 77.1 |
|  |  |  |  |  |  |  |

| Table S2. Model adequacy tests for private sector employees (n=7437) | | | | | | |
| --- | --- | --- | --- | --- | --- | --- |
| **Model selection**  **BIC / AIC** | **Group** | **n (%)** | **Posterior probability of group membership** | **Odds of correct classification** | **Probability observed** | **Probability expected** |
| **2-group** | 1 | 547 (7.4) | 99.9 | 15869.6 | 7.4 | 7.4 |
| BIC=632137.7 | 2 | 6890 (92.6) | 99.9 | 151.6 | 92.6 | 92.6 |
| AIC=632047.9 |  |  |  |  |  |  |
|  |  |  |  |  |  |  |
| **3-group** | 1 | 303 (4.1) | 99.8 | 9597.5 | 4.1 | 4.1 |
| BIC=531389.0 | 2 | 1037 (14.6) | 99.4 | 931.9 | 13.9 | 14.6 |
| AIC=531257.6 | 3 | 6097 (81.3) | 99.1 | 24.3 | 82.0 | 81.3 |
|  |  |  |  |  |  |  |
| **4-group** | 1 | 181 (2.4) | 99.4 | 7154.8 | 2.4 | 2.4 |
| BIC=483758.6 | 2 | 422 (5.9) | 99.0 | 1538.9 | 5.7 | 5.9 |
| AIC=483585.8 | 3 | 811 (11.4) | 98.6 | 538.9 | 10.9 | 11.4 |
|  | 4 | 6023 (80.2) | 98.9 | 23.1 | 81.0 | 80.2 |
|  |  |  |  |  |  |  |

| Table S3. Model adequacy tests for public sector employees (n=4378) | | | | | | |
| --- | --- | --- | --- | --- | --- | --- |
| **Model selection**  **BIC / AIC** | **Group** | **n (%)** | **Posterior probability of group membership** | **Odds of correct classification** | **Probability observed** | **Probability expected** |
| **2-group** | 1 | 452 (10.3) | 99.8 | 3774.2 | 10.3 | 10.3 |
| BIC=541656.3 | 2 | 3926 (89.7) | 100.0 | 247.1 | 89.7 | 89.7 |
| AIC=541573.3 |  |  |  |  |  |  |
|  |  |  |  |  |  |  |
| **3-group** | 1 | 196 (4.5) | 99.8 | 10908.0 | 4.5 | 4.5 |
| BIC=461758.5 | 2 | 692 (16.0) | 99.2 | 670.5 | 15.8 | 16.0 |
| AIC=461637.2 | 3 | 3490 (79.6) | 99.7 | 76.3 | 79.7 | 79.6 |
|  |  |  |  |  |  |  |
| **4-group** | 1 | 149 (3.4) | 100.0 | 87313.5 | 3.4 | 3.4 |
| BIC=423471.4 | 2 | 437 (10.0) | 98.0 | 430.0 | 10.0 | 10.0 |
| AIC=423311.8 | 3 | 452 (10.7) | 98.1 | 427.0 | 10.3 | 10.7 |
|  | 4 | 3340 (75.8) | 99.1 | 36.0 | 76.3 | 75.8 |
|  |  |  |  |  |  |  |
